# *N*,*N*-Diethyl-2-hy­droxy­ethanaminium 2,6-dioxo-5-(2,4,6-trinitro­phen­yl)-1,2,3,6-tetra­hydro­pyrimidin-4-olate dihydrate

**DOI:** 10.1107/S1600536811017363

**Published:** 2011-05-20

**Authors:** Manickam Buvaneswari, Doraisamyraja Kalaivani

**Affiliations:** a PG and Research Department of Chemistry, Seethalakshmi Ramaswami College, Tiruchirappalli 620 002, Tamil Nadu, India

## Abstract

In the title mol­ecular salt, C_6_H_16_NO^+^·C_10_H_4_N_5_O_9_^−^·2H_2_O, which crystallizes as a dihydrate, O—H⋯O hydrogen bonds link the barbiturate anion, the ethanaminium cation and the water mol­ecules of crystallization. The dihedral angle between the rings in the anion is 43.71 (8)°. In the crystal, an *R*_2_^2^(8) ring motif hydrogen-bonding pattern is also found involving inversion-related barbiturate rings with N—H⋯O hydrogen bonds. As a result of the various hydrogen bonds an infinite two-dimensional network, propagating in (10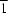), is formed.

## Related literature

For the anti-epileptic properties of barbiturates, see: Tripathi (2009 ▶); Kalaivani & Malarvizhi (2009 ▶); Kalaivani *et al.* (2008 ▶). For graph-set analysis of hydrogen bonds, see: Bernstein *et al.* (1995 ▶). 
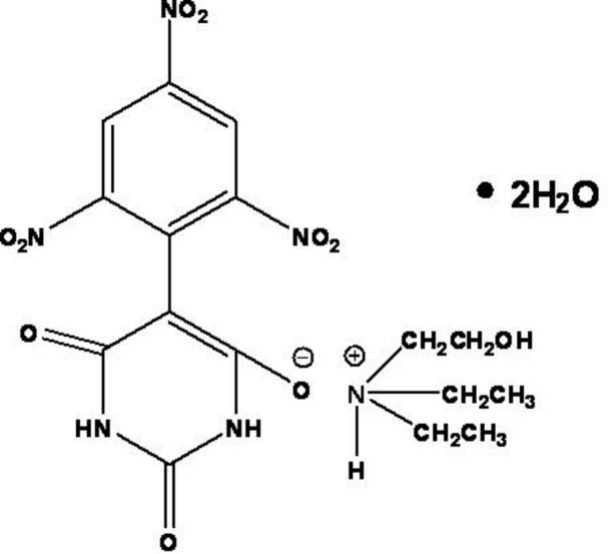


## Experimental

### 

#### Crystal data

C_6_H_16_NO^+^·C_10_H_4_N_5_O_9_^−^·2H_2_O
*M* *_r_* = 492.41
Monoclinic, 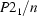

*a* = 8.3792 (2) Å
*b* = 21.7673 (4) Å
*c* = 12.0894 (2) Å
β = 96.118 (1)°
*V* = 2192.46 (8) Å^3^
*Z* = 4
Mo *K*α radiation
μ = 0.13 mm^−1^
*T* = 293 K
0.30 × 0.25 × 0.20 mm

#### Data collection

Bruker Kappa APEXII CCD diffractometer
Absorption correction: multi-scan (*SADABS*; Bruker 1999 ▶) *T*_min_ = 0.892, *T*_max_ = 0.975
23280 measured reflections
4686 independent reflections
3492 reflections with *I* > 2σ(*I*)
*R*_int_ = 0.037

#### Refinement

*R*[*F* ^2^ > 2σ(*F* ^2^)] = 0.042
*wR*(*F* ^2^) = 0.131
*S* = 1.08
4686 reflections
338 parameters
6 restraints
H atoms treated by a mixture of independent and constrained refinement
Δρ_max_ = 0.36 e Å^−3^
Δρ_min_ = −0.32 e Å^−3^

### 

Data collection: *APEX2* (Bruker, 2004 ▶); cell refinement: *SAINT-Plus* (Bruker, 2004 ▶); data reduction: *SAINT-Plus* and *XPREP* (Bruker, 2004 ▶); program(s) used to solve structure: *SIR92* (Altomare *et al.*, 1993 ▶); program(s) used to refine structure: *SHELXL97* (Sheldrick, 2008 ▶); molecular graphics: *ORTEP-3* (Farrugia, 1997 ▶) and *Mercury* (Macrae *et al.*, 2006 ▶); software used to prepare material for publication: *PLATON* (Spek, 2009 ▶).

## Supplementary Material

Crystal structure: contains datablocks global, I. DOI: 10.1107/S1600536811017363/su2264sup1.cif

Structure factors: contains datablocks I. DOI: 10.1107/S1600536811017363/su2264Isup2.hkl

Supplementary material file. DOI: 10.1107/S1600536811017363/su2264Isup3.cml

Additional supplementary materials:  crystallographic information; 3D view; checkCIF report

## Figures and Tables

**Table 1:** Hydrogen-bond geometry (Å, °)

*D*—H⋯*A*	*D*—H	H⋯*A*	*D*⋯*A*	*D*—H⋯*A*
O10—H10⋯O2*W*^i^	0.82	2.05	2.792 (2)	150
O10—H10⋯O4^ii^	0.82	2.49	3.0648 (19)	129
N6—H6⋯O1*W*^iii^	0.91 (3)	1.95 (3)	2.846 (2)	170 (2)
N1—H1*A*⋯O10^iv^	0.85 (2)	2.05 (3)	2.884 (2)	166 (2)
N2—H2*A*⋯O2^v^	0.83 (2)	2.03 (2)	2.847 (2)	173 (2)
O1*W*—H2*W*⋯O2^v^	0.91 (1)	1.90 (2)	2.7431 (19)	154 (2)
O2*W*—H4*W*⋯O1*W*^vi^	0.93 (1)	1.94 (2)	2.796 (3)	152 (3)
O1*W*—H1*W*⋯O3	0.91 (1)	1.91 (1)	2.7783 (17)	160 (2)
O2*W*—H3*W*⋯O1	0.93 (1)	1.89 (2)	2.792 (2)	162 (4)

Symmetry codes: (i) 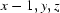; (ii) 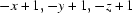; (iii) 
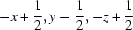
; (iv) 
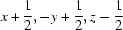
; (v) 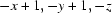; (vi) 
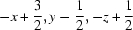
.

**Fig. 1.**
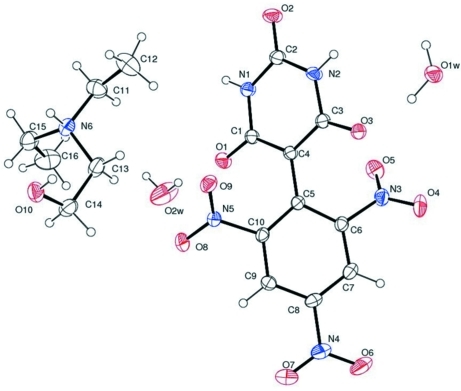
A view of the molecular structure of the title compound with the numbering scheme. Displacement ellipsoids are drawn at the 50% probability level.

**Fig. 2.**
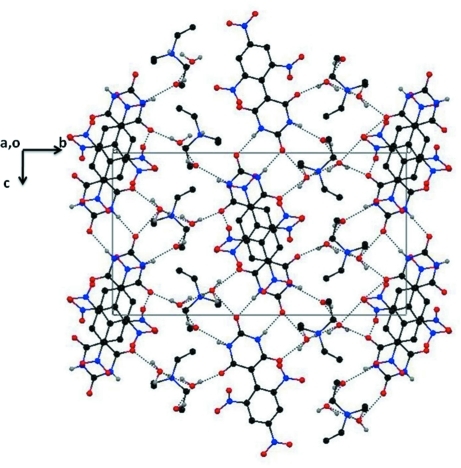
The crystal packing of the title compound viewed along the a axis. The O—H···O and N—H···O hydrogen bonds are shown as dashed lines (see Table 1 for details; C-bound H-atoms have been omitted for clarity).

## supplementary crystallographic information

### Comment

Most of the barbituric acid derivatives (barbiturates) are antiepileptic agents (Tripathi, 2009). The barbiturate prepared in our laboratory from 1-chloro-2,4-dinitrobenzene was obtained as maroon blocks when recrystallized from absolute alcohol (Kalaivani & Malarvizhi, 2009) and has antiepileptic activity (Kalaivani *et al.*, 2008). We report here the crystal structure of a related barbiturate obtained from 1-chloro-2,4,6-trinitrobenzene (TNCB) and barbituric acid in the presence of 2(*N,N*-diethyl) ethanolamine. It shows an extra ordinary stability and very high solubility in dipolar aprotic solvents, such as dimethyl sulfoxide, which may be attributed to its salt-like structure.

The molecular structure of the title compound is illustrated in Fig. 1. The anion and cation are involved in O—H···O hydrogen bonds with the water molecules of crystallization (Table 1).

In the crystal a *R*_2_^2^(8) ring motif hydrogen bonding pattern (Bernstein *et al.*, 1995) is also found involving inversion-related barbiturate rings with N—H···O hydrogen bonds (Table 1 and Fig. 2). These various hydrogen bonds lead finally to the formation of an infinite two-dimensional network propagating in (10–1).

### Experimental

Equimolar solutions of 1-chloro-2,4,6-trinitrobenzene (TNCB) and barbituric acid were prepared in ethanol and mixed. A three fold excess of 2(*N*,*N*-diethyl) ethanolamine was then added and the mixture was shaken well for 5–6 h. Maroon red coloured crystals were obtained after 24 hrs. The crystals were filtered and recrystallized from absolute alcohol (yield of pure crystals 70%, m.p. 507 K). Red block-like single crystals, suitable for X-ray diffraction analysis, were obtained by slow evaporation of an ethanolic solution of the title compound at room temperature.

### Refinement

The NH and water molecule H-atoms were located in difference electron density maps; the NH H-atoms were freely refined, while the water H-atoms were treated as riding atoms. The hydroxyl and C-bound H-atoms were included in calculated positions and treated as riding atoms: O—H = 0.82 Å, C—H = 0.93, 0.96, and 0.97 Å for CH(aromatic), CH_3_ and CH_2_ H-atoms, respectively, with *U*_iso_(H) = k × *U*_eq_(O,*C*) where k = 1.5 for CH_3_ H-atoms, and k = 1.2 for all other H-atoms.

### Crystal data

**Table:**

C_6_H_16_NO^+^·C_10_H_4_N_5_O_9_^−^·2H_2_O	*F*(000) = 1032
*M**_r_* = 492.41	*D*_x_ = 1.492 Mg m^−^^3^
Monoclinic, *P*2_1_/*n*	Mo *K*α radiation, λ = 0.71073 Å
Hall symbol: -P 2yn	Cell parameters from 5467 reflections
*a* = 8.3792 (2) Å	θ = 2.6–24.8°
*b* = 21.7673 (4) Å	µ = 0.13 mm^−^^1^
*c* = 12.0894 (2) Å	*T* = 293 K
β = 96.118 (1)°	Block, red
*V* = 2192.46 (8) Å^3^	0.30 × 0.25 × 0.20 mm
*Z* = 4	

### Data collection

**Table:**

Bruker Kappa APEXII CCD diffractometer	4686 independent reflections
Radiation source: fine-focus sealed tube	3492 reflections with *I* > 2σ(*I*)
graphite	*R*_int_ = 0.037
ω and φ scans	θ_max_ = 26.8°, θ_min_ = 1.9°
Absorption correction: multi-scan (*SADABS*; Bruker 1999)	*h* = −10→10
*T*_min_ = 0.892, *T*_max_ = 0.975	*k* = −27→27
23280 measured reflections	*l* = −15→15

### Refinement

**Table:**

Refinement on *F*^2^	Primary atom site location: structure-invariant direct methods
Least-squares matrix: full	Secondary atom site location: difference Fourier map
*R*[*F*^2^ > 2σ(*F*^2^)] = 0.042	Hydrogen site location: inferred from neighbouring sites
*wR*(*F*^2^) = 0.131	H atoms treated by a mixture of independent and constrained refinement
*S* = 1.08	*w* = 1/[σ^2^(*F*_o_^2^) + (0.0694*P*)^2^ + 0.4079*P*] where *P* = (*F*_o_^2^ + 2*F*_c_^2^)/3
4686 reflections	(Δ/σ)_max_ < 0.001
338 parameters	Δρ_max_ = 0.36 e Å^−^^3^
6 restraints	Δρ_min_ = −0.32 e Å^−^^3^

### Special details

**Table:**

Geometry. All e.s.d.'s (except the e.s.d. in the dihedral angle between two l.s. planes) are estimated using the full covariance matrix. The cell e.s.d.'s are taken into account individually in the estimation of e.s.d.'s in distances, angles and torsion angles; correlations between e.s.d.'s in cell parameters are only used when they are defined by crystal symmetry. An approximate (isotropic) treatment of cell e.s.d.'s is used for estimating e.s.d.'s involving l.s. planes.
Refinement. Refinement of *F*^2^ against ALL reflections. The weighted *R*-factor *wR* and goodness of fit *S* are based on *F*^2^, conventional *R*-factors *R* are based on *F*, with *F* set to zero for negative *F*^2^. The threshold expression of *F*^2^ > σ(*F*^2^) is used only for calculating *R*-factors(gt) *etc*. and is not relevant to the choice of reflections for refinement. *R*-factors based on *F*^2^ are statistically about twice as large as those based on *F*, and *R*- factors based on ALL data will be even larger.

### Fractional atomic coordinates and isotropic or equivalent isotropic displacement parameters (Å^2^)

**Table:**

	*x*	*y*	*z*	*U*_iso_*/*U*_eq_	
C1	0.7186 (2)	0.41169 (8)	0.30054 (14)	0.0306 (4)	
C2	0.5959 (2)	0.43691 (8)	0.11393 (15)	0.0388 (4)	
C3	0.6102 (2)	0.51490 (7)	0.25866 (14)	0.0300 (4)	
C4	0.6886 (2)	0.47250 (7)	0.33442 (14)	0.0292 (4)	
C5	0.73603 (19)	0.49194 (7)	0.44825 (13)	0.0266 (3)	
C6	0.81044 (19)	0.54867 (8)	0.47588 (14)	0.0302 (4)	
C7	0.8515 (2)	0.56930 (8)	0.58266 (15)	0.0356 (4)	
H7	0.8973	0.6079	0.5960	0.043*	
C8	0.8230 (2)	0.53139 (8)	0.66853 (15)	0.0350 (4)	
C9	0.7534 (2)	0.47444 (8)	0.65025 (15)	0.0327 (4)	
H9	0.7364	0.4487	0.7092	0.039*	
C10	0.70989 (19)	0.45705 (7)	0.54204 (14)	0.0286 (4)	
C11	0.4444 (3)	0.22119 (12)	0.27523 (19)	0.0554 (6)	
H11A	0.5560	0.2339	0.2822	0.067*	
H11B	0.4334	0.1863	0.2251	0.067*	
C12	0.3427 (4)	0.27264 (14)	0.2257 (2)	0.0765 (8)	
H12A	0.2315	0.2631	0.2295	0.115*	
H12B	0.3616	0.2782	0.1494	0.115*	
H12C	0.3695	0.3098	0.2664	0.115*	
C13	0.4327 (3)	0.25114 (9)	0.47133 (18)	0.0488 (5)	
H13A	0.3762	0.2878	0.4436	0.059*	
H13B	0.5466	0.2604	0.4794	0.059*	
C14	0.3828 (3)	0.23605 (10)	0.58351 (17)	0.0475 (5)	
H14A	0.4519	0.2039	0.6172	0.057*	
H14B	0.3969	0.2721	0.6307	0.057*	
C15	0.4762 (3)	0.14050 (9)	0.42105 (19)	0.0504 (5)	
H15A	0.4378	0.1276	0.4903	0.060*	
H15B	0.4401	0.1103	0.3648	0.060*	
C16	0.6552 (3)	0.14057 (14)	0.4358 (2)	0.0731 (8)	
H16A	0.6949	0.1503	0.3663	0.110*	
H16B	0.6933	0.1007	0.4602	0.110*	
H16C	0.6926	0.1707	0.4905	0.110*	
N1	0.6672 (2)	0.39757 (7)	0.19009 (13)	0.0372 (4)	
N2	0.5695 (2)	0.49417 (7)	0.15108 (13)	0.0399 (4)	
N3	0.85714 (19)	0.59043 (7)	0.38903 (14)	0.0390 (4)	
N4	0.8702 (2)	0.55069 (9)	0.78267 (15)	0.0504 (4)	
N5	0.62095 (18)	0.39864 (6)	0.53118 (12)	0.0323 (3)	
N6	0.4013 (2)	0.20141 (7)	0.38774 (14)	0.0391 (4)	
O1	0.78610 (16)	0.37089 (6)	0.35927 (10)	0.0405 (3)	
O2	0.5564 (2)	0.42190 (6)	0.01670 (11)	0.0583 (4)	
O1W	0.42307 (17)	0.66131 (6)	0.15158 (11)	0.0433 (3)	
O3	0.57236 (15)	0.56815 (5)	0.28132 (10)	0.0351 (3)	
O2W	0.9751 (3)	0.27123 (10)	0.4379 (2)	0.1173 (10)	
O4	0.8348 (2)	0.64511 (6)	0.40147 (14)	0.0615 (4)	
O5	0.92002 (16)	0.56847 (7)	0.31178 (12)	0.0482 (4)	
O6	0.9235 (3)	0.60220 (9)	0.79794 (14)	0.0794 (6)	
O7	0.8528 (3)	0.51453 (9)	0.85712 (14)	0.0882 (7)	
O8	0.66984 (18)	0.35673 (6)	0.59242 (12)	0.0486 (4)	
O9	0.50041 (15)	0.39638 (6)	0.46545 (11)	0.0408 (3)	
O10	0.22086 (17)	0.21639 (6)	0.57806 (12)	0.0449 (3)	
H10	0.1618	0.2438	0.5511	0.071 (8)*	
H6	0.294 (3)	0.1935 (11)	0.377 (2)	0.063 (7)*	
H1A	0.686 (3)	0.3612 (11)	0.1685 (19)	0.052 (6)*	
H2A	0.526 (3)	0.5191 (10)	0.1057 (18)	0.044 (6)*	
H1W	0.467 (3)	0.6368 (9)	0.2074 (13)	0.071 (8)*	
H2W	0.424 (4)	0.6446 (11)	0.0830 (10)	0.091 (10)*	
H4W	0.978 (5)	0.2365 (12)	0.393 (3)	0.162 (18)*	
H3W	0.895 (4)	0.2983 (15)	0.411 (3)	0.160 (17)*	

### Atomic displacement parameters (Å^2^)

**Table:**

	*U*^11^	*U*^22^	*U*^33^	*U*^12^	*U*^13^	*U*^23^
C1	0.0348 (9)	0.0271 (8)	0.0296 (9)	0.0051 (7)	0.0016 (7)	−0.0002 (7)
C2	0.0517 (11)	0.0312 (9)	0.0317 (9)	0.0083 (8)	−0.0043 (8)	−0.0032 (8)
C3	0.0314 (8)	0.0280 (8)	0.0301 (9)	0.0034 (7)	0.0004 (7)	−0.0016 (7)
C4	0.0326 (9)	0.0261 (8)	0.0279 (8)	0.0042 (7)	−0.0011 (7)	−0.0002 (7)
C5	0.0244 (8)	0.0238 (8)	0.0306 (8)	0.0049 (6)	−0.0014 (6)	−0.0006 (6)
C6	0.0292 (8)	0.0260 (8)	0.0347 (9)	0.0010 (6)	−0.0005 (7)	0.0006 (7)
C7	0.0334 (9)	0.0296 (9)	0.0425 (10)	−0.0024 (7)	−0.0016 (7)	−0.0068 (8)
C8	0.0330 (9)	0.0385 (10)	0.0318 (9)	0.0007 (7)	−0.0046 (7)	−0.0087 (8)
C9	0.0334 (9)	0.0329 (9)	0.0306 (9)	0.0022 (7)	−0.0021 (7)	0.0028 (7)
C10	0.0282 (8)	0.0234 (8)	0.0332 (9)	0.0010 (6)	−0.0017 (6)	−0.0008 (7)
C11	0.0560 (13)	0.0660 (15)	0.0457 (12)	−0.0002 (11)	0.0114 (10)	0.0030 (11)
C12	0.0832 (19)	0.085 (2)	0.0612 (16)	0.0084 (15)	0.0074 (14)	0.0235 (14)
C13	0.0556 (13)	0.0366 (10)	0.0552 (13)	−0.0108 (9)	0.0103 (10)	−0.0117 (9)
C14	0.0560 (13)	0.0404 (11)	0.0445 (11)	−0.0031 (9)	−0.0013 (9)	−0.0096 (9)
C15	0.0569 (13)	0.0376 (11)	0.0558 (13)	0.0069 (9)	0.0021 (10)	−0.0051 (9)
C16	0.0578 (15)	0.0869 (19)	0.0718 (17)	0.0213 (14)	−0.0061 (12)	−0.0082 (15)
N1	0.0529 (10)	0.0254 (8)	0.0319 (8)	0.0097 (7)	−0.0019 (7)	−0.0048 (6)
N2	0.0599 (11)	0.0290 (8)	0.0278 (8)	0.0144 (7)	−0.0094 (7)	−0.0010 (6)
N3	0.0379 (8)	0.0326 (8)	0.0460 (9)	−0.0053 (6)	0.0016 (7)	0.0030 (7)
N4	0.0570 (11)	0.0543 (11)	0.0378 (9)	−0.0070 (9)	−0.0040 (8)	−0.0117 (8)
N5	0.0382 (8)	0.0256 (7)	0.0328 (8)	−0.0008 (6)	0.0016 (6)	0.0000 (6)
N6	0.0398 (9)	0.0343 (8)	0.0432 (9)	−0.0031 (7)	0.0049 (7)	−0.0058 (7)
O1	0.0522 (8)	0.0303 (6)	0.0374 (7)	0.0153 (6)	−0.0020 (6)	0.0024 (5)
O2	0.0989 (13)	0.0384 (8)	0.0327 (7)	0.0199 (8)	−0.0160 (7)	−0.0091 (6)
O1W	0.0544 (8)	0.0367 (7)	0.0372 (8)	0.0126 (6)	−0.0024 (6)	0.0005 (6)
O3	0.0445 (7)	0.0252 (6)	0.0340 (6)	0.0100 (5)	−0.0029 (5)	−0.0019 (5)
O2W	0.142 (2)	0.0625 (13)	0.128 (2)	0.0511 (14)	−0.0737 (17)	−0.0340 (13)
O4	0.0903 (13)	0.0250 (7)	0.0697 (11)	−0.0070 (7)	0.0110 (9)	0.0037 (7)
O5	0.0441 (8)	0.0523 (8)	0.0502 (8)	0.0017 (6)	0.0148 (7)	0.0066 (7)
O6	0.1110 (16)	0.0719 (12)	0.0536 (10)	−0.0391 (11)	0.0006 (10)	−0.0260 (9)
O7	0.150 (2)	0.0752 (13)	0.0342 (9)	−0.0199 (13)	−0.0119 (10)	−0.0011 (9)
O8	0.0660 (10)	0.0280 (7)	0.0497 (8)	−0.0008 (6)	−0.0039 (7)	0.0100 (6)
O9	0.0360 (7)	0.0376 (7)	0.0469 (8)	−0.0067 (5)	−0.0036 (6)	−0.0036 (6)
O10	0.0520 (8)	0.0322 (7)	0.0504 (8)	−0.0001 (6)	0.0049 (6)	0.0069 (6)

### Geometric parameters (Å, °)

**Table:**

C1—O1	1.236 (2)	C13—N6	1.485 (2)
C1—N1	1.393 (2)	C13—C14	1.497 (3)
C1—C4	1.416 (2)	C13—H13A	0.9700
C2—O2	1.231 (2)	C13—H13B	0.9700
C2—N1	1.350 (2)	C14—O10	1.418 (3)
C2—N2	1.351 (2)	C14—H14A	0.9700
C3—O3	1.240 (2)	C14—H14B	0.9700
C3—N2	1.385 (2)	C15—C16	1.492 (3)
C3—C4	1.412 (2)	C15—N6	1.503 (3)
C4—C5	1.454 (2)	C15—H15A	0.9700
C5—C10	1.401 (2)	C15—H15B	0.9700
C5—C6	1.407 (2)	C16—H16A	0.9600
C6—C7	1.376 (2)	C16—H16B	0.9600
C6—N3	1.473 (2)	C16—H16C	0.9600
C7—C8	1.367 (3)	N1—H1A	0.85 (2)
C7—H7	0.9300	N2—H2A	0.83 (2)
C8—C9	1.378 (2)	N3—O4	1.217 (2)
C8—N4	1.456 (2)	N3—O5	1.218 (2)
C9—C10	1.373 (2)	N4—O6	1.214 (2)
C9—H9	0.9300	N4—O7	1.216 (2)
C10—N5	1.472 (2)	N5—O9	1.2177 (19)
C11—C12	1.493 (3)	N5—O8	1.2180 (18)
C11—N6	1.507 (3)	N6—H6	0.91 (3)
C11—H11A	0.9700	O1W—H1W	0.908 (9)
C11—H11B	0.9700	O1W—H2W	0.907 (9)
C12—H12A	0.9600	O2W—H4W	0.933 (10)
C12—H12B	0.9600	O2W—H3W	0.929 (10)
C12—H12C	0.9600	O10—H10	0.8200
			
O1—C1—N1	117.90 (15)	N6—C13—H13B	108.7
O1—C1—C4	126.17 (15)	C14—C13—H13B	108.7
N1—C1—C4	115.93 (14)	H13A—C13—H13B	107.6
O2—C2—N1	122.52 (17)	O10—C14—C13	112.47 (17)
O2—C2—N2	121.69 (17)	O10—C14—H14A	109.1
N1—C2—N2	115.79 (16)	C13—C14—H14A	109.1
O3—C3—N2	117.98 (15)	O10—C14—H14B	109.1
O3—C3—C4	125.44 (15)	C13—C14—H14B	109.1
N2—C3—C4	116.56 (15)	H14A—C14—H14B	107.8
C3—C4—C1	120.54 (15)	C16—C15—N6	114.6 (2)
C3—C4—C5	119.01 (14)	C16—C15—H15A	108.6
C1—C4—C5	120.44 (14)	N6—C15—H15A	108.6
C10—C5—C6	112.77 (14)	C16—C15—H15B	108.6
C10—C5—C4	123.89 (15)	N6—C15—H15B	108.6
C6—C5—C4	123.33 (15)	H15A—C15—H15B	107.6
C7—C6—C5	124.70 (16)	C15—C16—H16A	109.5
C7—C6—N3	114.12 (15)	C15—C16—H16B	109.5
C5—C6—N3	121.13 (15)	H16A—C16—H16B	109.5
C8—C7—C6	117.98 (16)	C15—C16—H16C	109.5
C8—C7—H7	121.0	H16A—C16—H16C	109.5
C6—C7—H7	121.0	H16B—C16—H16C	109.5
C7—C8—C9	121.78 (16)	C2—N1—C1	125.68 (15)
C7—C8—N4	119.59 (16)	C2—N1—H1A	117.5 (15)
C9—C8—N4	118.62 (17)	C1—N1—H1A	116.8 (15)
C10—C9—C8	117.76 (16)	C2—N2—C3	125.47 (16)
C10—C9—H9	121.1	C2—N2—H2A	117.4 (15)
C8—C9—H9	121.1	C3—N2—H2A	117.1 (15)
C9—C10—C5	124.95 (15)	O4—N3—O5	124.29 (17)
C9—C10—N5	113.79 (15)	O4—N3—C6	117.36 (16)
C5—C10—N5	121.14 (14)	O5—N3—C6	118.30 (15)
C12—C11—N6	113.3 (2)	O6—N4—O7	123.81 (18)
C12—C11—H11A	108.9	O6—N4—C8	117.96 (19)
N6—C11—H11A	108.9	O7—N4—C8	118.22 (17)
C12—C11—H11B	108.9	O9—N5—O8	124.63 (15)
N6—C11—H11B	108.9	O9—N5—C10	118.02 (14)
H11A—C11—H11B	107.7	O8—N5—C10	117.27 (14)
C11—C12—H12A	109.5	C13—N6—C15	114.97 (17)
C11—C12—H12B	109.5	C13—N6—C11	111.34 (16)
H12A—C12—H12B	109.5	C15—N6—C11	111.13 (17)
C11—C12—H12C	109.5	C13—N6—H6	109.8 (16)
H12A—C12—H12C	109.5	C15—N6—H6	104.4 (16)
H12B—C12—H12C	109.5	C11—N6—H6	104.5 (16)
N6—C13—C14	114.21 (16)	H1W—O1W—H2W	113.6 (14)
N6—C13—H13A	108.7	H4W—O2W—H3W	111.6 (16)
C14—C13—H13A	108.7	C14—O10—H10	109.5
			
O3—C3—C4—C1	176.75 (17)	N6—C13—C14—O10	−53.0 (2)
N2—C3—C4—C1	−1.6 (2)	O2—C2—N1—C1	178.4 (2)
O3—C3—C4—C5	−2.4 (3)	N2—C2—N1—C1	−2.0 (3)
N2—C3—C4—C5	179.24 (16)	O1—C1—N1—C2	−178.03 (18)
O1—C1—C4—C3	179.91 (17)	C4—C1—N1—C2	1.6 (3)
N1—C1—C4—C3	0.3 (2)	O2—C2—N2—C3	−179.9 (2)
O1—C1—C4—C5	−1.0 (3)	N1—C2—N2—C3	0.4 (3)
N1—C1—C4—C5	179.45 (15)	O3—C3—N2—C2	−177.22 (18)
C3—C4—C5—C10	134.25 (17)	C4—C3—N2—C2	1.3 (3)
C1—C4—C5—C10	−44.9 (2)	C7—C6—N3—O4	−42.6 (2)
C3—C4—C5—C6	−44.4 (2)	C5—C6—N3—O4	139.73 (18)
C1—C4—C5—C6	136.50 (17)	C7—C6—N3—O5	135.05 (17)
C10—C5—C6—C7	−1.2 (2)	C5—C6—N3—O5	−42.6 (2)
C4—C5—C6—C7	177.56 (16)	C7—C8—N4—O6	5.1 (3)
C10—C5—C6—N3	176.24 (15)	C9—C8—N4—O6	−176.2 (2)
C4—C5—C6—N3	−5.0 (2)	C7—C8—N4—O7	−175.4 (2)
C5—C6—C7—C8	2.2 (3)	C9—C8—N4—O7	3.3 (3)
N3—C6—C7—C8	−175.40 (16)	C9—C10—N5—O9	133.96 (16)
C6—C7—C8—C9	−0.9 (3)	C5—C10—N5—O9	−42.3 (2)
C6—C7—C8—N4	177.78 (16)	C9—C10—N5—O8	−42.9 (2)
C7—C8—C9—C10	−1.3 (3)	C5—C10—N5—O8	140.79 (16)
N4—C8—C9—C10	−179.93 (16)	C14—C13—N6—C15	−56.2 (2)
C8—C9—C10—C5	2.4 (3)	C14—C13—N6—C11	176.34 (19)
C8—C9—C10—N5	−173.72 (15)	C16—C15—N6—C13	−64.0 (3)
C6—C5—C10—C9	−1.2 (2)	C16—C15—N6—C11	63.6 (2)
C4—C5—C10—C9	−179.92 (16)	C12—C11—N6—C13	−64.7 (3)
C6—C5—C10—N5	174.67 (14)	C12—C11—N6—C15	165.7 (2)
C4—C5—C10—N5	−4.1 (2)		

### Hydrogen-bond geometry (Å, °)

**Table:**

*D*—H···*A*	*D*—H	H···*A*	*D*···*A*	*D*—H···*A*
O10—H10···O2W^i^	0.82	2.05	2.792 (2)	150
O10—H10···O4^ii^	0.82	2.49	3.0648 (19)	129
N6—H6···O1W^iii^	0.91 (3)	1.95 (3)	2.846 (2)	170 (2)
N1—H1A···O10^iv^	0.85 (2)	2.05 (3)	2.884 (2)	166 (2)
N2—H2A···O2^v^	0.83 (2)	2.03 (2)	2.847 (2)	173 (2)
O1W—H2W···O2^v^	0.91 (1)	1.90 (2)	2.7431 (19)	154 (2)
O2W—H4W···O1W^vi^	0.93 (1)	1.94 (2)	2.796 (3)	152 (3)
O1W—H1W···O3	0.91 (1)	1.91 (1)	2.7783 (17)	160 (2)
O2W—H3W···O1	0.93 (1)	1.89 (2)	2.792 (2)	162 (4)

Symmetry codes: (i) *x*−1, *y*, *z*; (ii) −*x*+1, −*y*+1, −*z*+1; (iii) −*x*+1/2, *y*−1/2, −*z*+1/2; (iv) *x*+1/2, −*y*+1/2, *z*−1/2; (v) −*x*+1, −*y*+1, −*z*; (vi) −*x*+3/2, *y*−1/2, −*z*+1/2.
